# New Xerophilic Species of *Penicillium* from Soil

**DOI:** 10.3390/jof7020126

**Published:** 2021-02-09

**Authors:** Ernesto Rodríguez-Andrade, Alberto M. Stchigel, José F. Cano-Lira

**Affiliations:** Mycology Unit, Medical School and IISPV, Universitat Rovira i Virgili (URV), Sant Llorenç 21, Reus, 43201 Tarragona, Spain; netorock_11@hotmail.com (E.R.-A.); jose.cano@urv.cat (J.F.C.-L.)

**Keywords:** Ascomycota, Eurotiales, *Penicillium*, phylogeny, soil, taxonomy, xerophilic

## Abstract

Soil is one of the main reservoirs of fungi. The aim of this study was to study the richness of ascomycetes in a set of soil samples from Mexico and Spain. Fungi were isolated after 2% *w*/*v* phenol treatment of samples. In that way, several strains of the genus *Penicillium* were recovered. A phylogenetic analysis based on internal transcribed spacer (ITS), beta-tubulin (*BenA*), calmodulin (*CaM*), and RNA polymerase II subunit 2 gene (*rpb*2) sequences showed that four of these strains had not been described before. *Penicillium melanosporum* produces monoverticillate conidiophores and brownish conidia covered by an ornate brown sheath. *Penicillium michoacanense* and *Penicillium siccitolerans* produce sclerotia, and their asexual morph is similar to species in the section *Aspergilloides* (despite all of them pertaining to section *Lanata*-*Divaricata*). *P. michoacanense* differs from *P. siccitolerans* in having thick-walled peridial cells (thin-walled in *P. siccitolerans*). *Penicillium sexuale* differs from *Penicillium cryptum* in the section *Crypta* because it does not produce an asexual morph. Its ascostromata have a peridium composed of thick-walled polygonal cells, and its ascospores are broadly lenticular with two equatorial ridges widely separated by a furrow. All four new species are xerophilic. Despite the genus *Penicillium* containing more than 480 known species, they are rarely reported as xerophilic.

## 1. Introduction

Soil is the natural reservoir of numerous organisms, such as algae, archaea, bacteria, fungi, protozoa, helminths, and arthropods, forming populations that are in a dynamic ecological balance. Many of these are responsible for degrading dead plants and animal remains to less complex molecules and contribute to the formation of humus and the maintenance of soil fertility [1]. Fungi grow in the space between soil particles, and nutrients necessary for their development are provided by organic matter and/or living roots. After early colonizers removed most of the soluble nutrients, fungi attack any remaining insoluble substrates by producing a broad spectrum of hydrolytic enzymes [2]. The products of enzymatic degradation are readily available, with various organisms competing for the same ecological niche. In addition, many of these fungi produce substances with antibiotic activity, which help them to compete more effectively for the scarcely available nutritional resources. Among them, species of the genus *Penicillium* are always present. The genus *Penicillium* was described by Link [3] in order to place three previously unknown fungi (*Penicillium candidum*, *Penicillium glaucum,* and *Penicillium expansum*, the latter corresponding to the type species of the genus). They typically produce brush-like structures (conidiophores) responsible for the formation of the asexual spores (conidia). At present, more than 480 species of *Penicillium* have been described [4,5] and more than 1300 records are available (Index of Fungi; http://www.indexfungorum.org/Names/Names.asp; 18 February 2021). Soil-borne *Penicillium* species are ubiquitous and present in a wide range of environmental conditions [4]. Several of them are extremophiles, meaning they can proliferate in low or high temperatures, in low pH values, and in high salt or sugar concentrations [6,7,8,9,10,11]. Only a few species have been reported as xerophilic, and consequently able to grow at a water activity (a_w_) of 0.85 or below [12].

The aim of the present study was to resolve the taxonomic position and to identify several xerophilic isolates of *Penicillium* from soils in Spain and Mexico based on their morphology and a multilocus phylogenetic analysis (internal transcribed spacer (ITS), beta-tubulin (*BenA*), calmodulin (*CaM*), and RNA polymerase II subunit 2 gene (*rpb*2)).

## 2. Materials and Methods

### 2.1. Sampling and Fungal Isolation

Soil samples were collected near the small villages of El Zapote (Michoacán state, Mexico) and Riaza (Castilla y León community, Spain). Riaza (41°16′59″ N, 3°28′00″ W) is at 1190 m above sea level (MASL), with a Mediterranean cold summer climate (according to the Köppen–Gieger climate classification), the average annual temperature is between 8 °C and 12 °C, the average annual rainfall is above 700 mm, and soils are based on metamorphic slate, quartzite, and schist, and covered with oaks (*Quercus pyrenaica*) trees. El Zapote (19°57′14.9” N, 101°38′34.8” W) is at 2100 MASL., with a dry-winter subtropical highland climate, the average annual temperature is 16–18 °C, the average annual rainfall is between 800 mm and 1000 mm, and soils are of extrusive volcanic origin, the vast majority of which are used to cultivate corn and sorghum. Samples from a horizon free from recognizable organic matter were placed into sterile plastic bags, which were then sealed. Once in the laboratory, the samples were stored at room temperature in the dark until they were processed. The methodology used for fungal isolation is described in Stchigel et al. [13]. Briefly, 1 g of soil was placed into a test tube, mixed with 5 mL 2% (*w*/*v*) phenol (Panreac, Barcelona, Spain) by shaking, and left to settle for 10 min. Then, the supernatant was discarded and the sediment was resuspended in 10 mL of sterilized water. The suspensions (1.6 mL) were poured into Petri dishes and mixed with 15 mL of molten (at 50–55 °C) sterile potato-carrot agar medium (PCA; potatoes, 20 g; carrot, 20 g, 1 L tap water). After jellification of the culture medium, Petri dishes were incubated at room temperature (22–25 °C) in the dark until 4–5 weeks. Cultures were examined periodically under a stereomicroscope and, when the formation of reproductive structures was observed, they were transferred to Petri dishes containing potato dextrose agar (PDA; Pronadisa, Madrid, Spain) [14] supplemented with L-chloramphenicol (100 mg/L), and incubated at 22–25 °C in the dark.

### 2.2. Phenotypic Study

Cultural characterization was carried out following the recommendations of Visagie et al. [4]. Briefly, spores suspensions in a semi-solid medium (0.2% agar; 0.05% Tween 80; homemade) were inoculated into 90-mm diameter Petri dishes at three equidistant points onto malt extract agar (MEA; Difco, Detroit, MI, USA) [15], oatmeal agar (OA; homemade) [15], Czapek yeast extract agar (CYA; homemade) [16], yeast extract sucrose agar (YES; homemade) [17], creatine sucrose agar (CREA; homemade) [17], dichloran^®^ 18% glycerol agar (DG18; homemade) [18], 25% glycerol nitrate agar (G25N; homemade), [19] and malt extract yeast extract 70% fructose-glucose (MY70FG; homemade) [20] and then grown at 25 °C for 14 days in the dark. To determine the minimum, optimum, and maximum temperatures of growth, colony diameters were measured after 14 days at 5, 15, 25, 30, 37, and 40 °C on CYA. Color notations were according to Kornerup and Wanscher [21]. Microscopic characterization was carried out after 14 days of growth on MEA at 25 °C and the fungal structures were mounted on a drop of lactophenol between the slide and cover slide. Photomicrographs were taken using a Zeiss Axio-Imager M1 bright field microscope (Oberkochen, Germany) with a DeltaPix Infinity X digital camera, with Nomarski differential interference contrast and phase contrast optics. The novel taxonomic descriptions and the proposed names were deposited in MycoBank (http://www.mycobank.org; 18 June 2020) [22].

### 2.3. DNA Extraction, Amplification, and Sequencing

Total DNA was extracted directly from colonies on MEA after 7–10 days incubation at 25 °C in the dark, following the Fast DNA kit protocol (Bio 101, Inc., Vista, CA, USA) with the homogenization step repeated three times with a FastPrep FP120 instrument (Thermo Savant, Holbrook, NY, USA). After each homogenization, the sample was kept in ice for 10 min. DNA was quantified with GeneQuant pro (Amersham Pharmacia Biotech, Cambridge, England) [23]. Extracted DNA was used to amplify the internal transcribed spacer (ITS) (ITS5/ITS4 primers) [24], a fragment of the beta-tubulin (*BenA*) (T10/Bt2b primers) [25], a fragment of the calmodulin (*CaM*) (Cmd5/Cmd6 primers) [26] and a fragment of the RNA polymerase II subunit 2 gene (*rpb*2) (RPB2-5F/RPB2-7cR primers) [27]. The PCR amplifications were made in a total volume of 25 μL containing 5 μL 10x PCR Buffer (Invitrogen, CA, USA), 0.2 μM dNTPs, 0.5 μM of each primer, 1 U Taq DNA polymerase, and 1–10 ng nuclear DNA. PCR conditions for ITS, *BenA*, *CaM,* and *rpb*2 were set as follows: initial denaturation at 95 °C for 5 min, followed by 35 cycles of denaturation, annealing and extension, and a final extension step at 72 °C for 10 min. For ITS amplification, the 35 cycles were 45 s at 95 °C, 45 s at 53 °C, and 2 min at 72 °C; for the *BenA* region, 30 s at 95 °C, 1 min at 55 °C, and 90 s at 72 °C; for *CaM* region, 30 s at 94 °C, 1 min at 55 °C, and 90 s at 72 °C; and for the *rpb*2 region, 45 s at 95 °C, 1 min at 56 °C, and 90 s at 72 °C. Single-band PCR products were purified from agarose gels and sequenced at Macrogen Europe, which uses large-scale sequencing developed by “Applied Biosystem,” which works using the Sanger method (Macrogen Inc., Madrid, Spain). Sequence assembly and editing were carried out using SeqMan software v. 7.0 (DNAStar Lasergene, Madison, WI, USA). GenBank accession numbers for the newly generated sequences in this study and others corresponding to reference or ex-type strains are listed in Supplementary Table S1.

### 2.4. Phylogenetic Analysis

**Figure 1:**
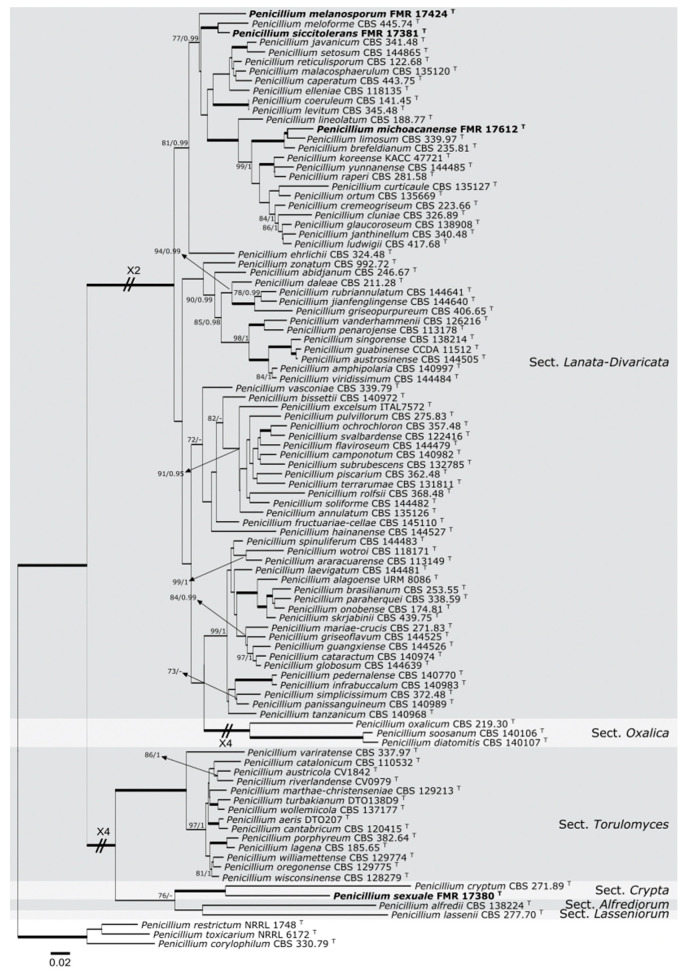
Maximum-likelihood (ML) phylogenetic tree of *Penicillium* section *Alfrediorum*, *Crypta*, *Lanata-Divaricata*, *Lasseniorum*, *Oxalica, and Torulomyces* inferred from the combined internal transcribed spacer (ITS), beta-tubulin (*BenA*), calmodulin (*CaM*), and RNA polymerase II subunit 2 gene (*rpb*2) loci. Support in nodes is indicated above thick branches and is represented by posterior probabilities (Bayesian inference (BI) analysis) of 0.95 and higher and/or bootstrap values (ML analysis) of 70% and higher. Some branches were shortened; these are indicated by two diagonal lines with the number of times a branch was shortened. Fully supported branched (100% BS/1 PP) are indicated in **bold**. ^T^ = ex-type strains. Alignment length 2249 bp. The sequences not generated by us were retrieved from EMBL/GenBank and are indicated in Supplementary Table S1.

The sequences of the four loci generated in this study were compared with those of the National Center for Biotechnology Information using the Basic Local Alignment Search Tool (BLAST; https://blast.ncbi.nlm.nih.gov/Blast.cgi?PROGRAM=blastn&PAGE_TYPE=BlastSearch&LINK_LOC=blasthome; 10 July 2019). To determine the phylogenetic relationship of all isolates, a combination of ITS–*BenA*–*CaM*–*rpb*2 was built to distinguish among other species of *Penicillium* belong to the sections *Alfrediorum*, *Crypta*, *Lanata-Divaricata*, *Lasseniorum*, *Oxalica*, and *Torulomyces* (Figure 1). *Penicillium toxicarium* NRRL 6172, *Penicillium restrictum* NRRL 1748, and *Penicillium corylophilum* CBS 330.79 (section *Exilicaulis*) were selected as outgroups. The sequence alignments and the maximum-likelihood (ML) and Bayesian inference (BI) phylogenetic analyses were performed as was described by Valenzuela-Lopez et al. [28]. The final matrices used for phylogenetic analyses were deposited in TreeBASE (www.treebase.org; accession number: 25066; 12 July 2019).

## 3. Results

### 3.1. Molecular Phylogeny

The Blast search gave the following results: strain FMR 17,424 matched with *Penicillium meloforme* CBS 445.74 (similarity: ITS, 97%; *BenA*, 93.3%; *CaM*, 88.2%; *rpb*2, 93.9%); FMR 17,381 with *P. meloforme* CBS 445.74 (similarity: ITS, 99.2%; BenA, 98.1%; CaM, 96.8%; rpb2, 98.8%); FMR 17,612 with *P. limosum* CBS 339.97 (similarity: ITS, 99.6%; BenA, 97.2%; CaM, 96.3%; rpb2, 99%); and FMR 17,380 with *P. wisconsinense* CBS 128,279 (similarity: ITS, 94%) and with *P. cryptum* CBS 271.89 (similarity: BenA, 86%; CaM, 85.6%; rpb2, 89.7%).

We carried out individual and combined phylogenetic analyses with ITS, *BenA*, *CaM*, and *rpb*2 sequences to resolve the taxonomical position of our strains using the sequences of type strains of the accepted species of *Penicillium* into the sections *Lanata*-*Divaricata* and *Torulomyces*. A concatenated dataset from 98 sequences contained a total of 2249 characters including gaps (575 of them for ITS, 444 for *BenA*, 476 for *CaM*, and 755 for *rpb*2), from which 966 were parsimony informative (114 for ITS, 257 for *BenA*, 310 for *CaM*, and 285 for *rpb*2). The sequence datasets did not show conflict in the tree topologies for the 70% reciprocal bootstrap trees, which allowed us to combine the four genes for the multi-locus analysis. The ML analysis showed similar tree topology and was congruent with that obtained in the Bayesian inference analysis. The phylogenetic tree (Figure 1) was divided into six main clades representing the sections *Alfrediorum*, *Crypta* (100% BS/1 PP), *Lanata*-*Divaricata* (100% BS/1 PP), *Lasseniorum*, *Oxalica* (100% BS/1 PP), and *Torulomyces* (100% BS/1 PP). Three of our strains were placed into the section *Lanata*-*Divaricata* clade—FMR 17,424, which formed a sister branch of a terminal clade; FMR 17,381, which formed in another terminal clade containing *P. meloforme* CBS 445.74 (100% BS/1 PP); and FMR 17,612, which formed a terminal clade (100% BS/1 PP) with *P. brefeldianum* CBS 235.81 and *P. limosum* CBS 339.97. On the other hand, strain FMR 17,380 was located in the section *Crypta*, into a terminal clade (100% BS/1 PP), together with *P. cryptum*.

### 3.2. Taxonomy

Because FMR 17,424 forms a sister branch distant from the nearest terminal clade composed by FMR 17,381 and *P. meloforme*, and because FMR 17,381 differs phylogenetically and phenotypically from mentioned species, both strains are proposed as two new species of section *Lanata-Divaricata* as follows.

**Figure 2:**
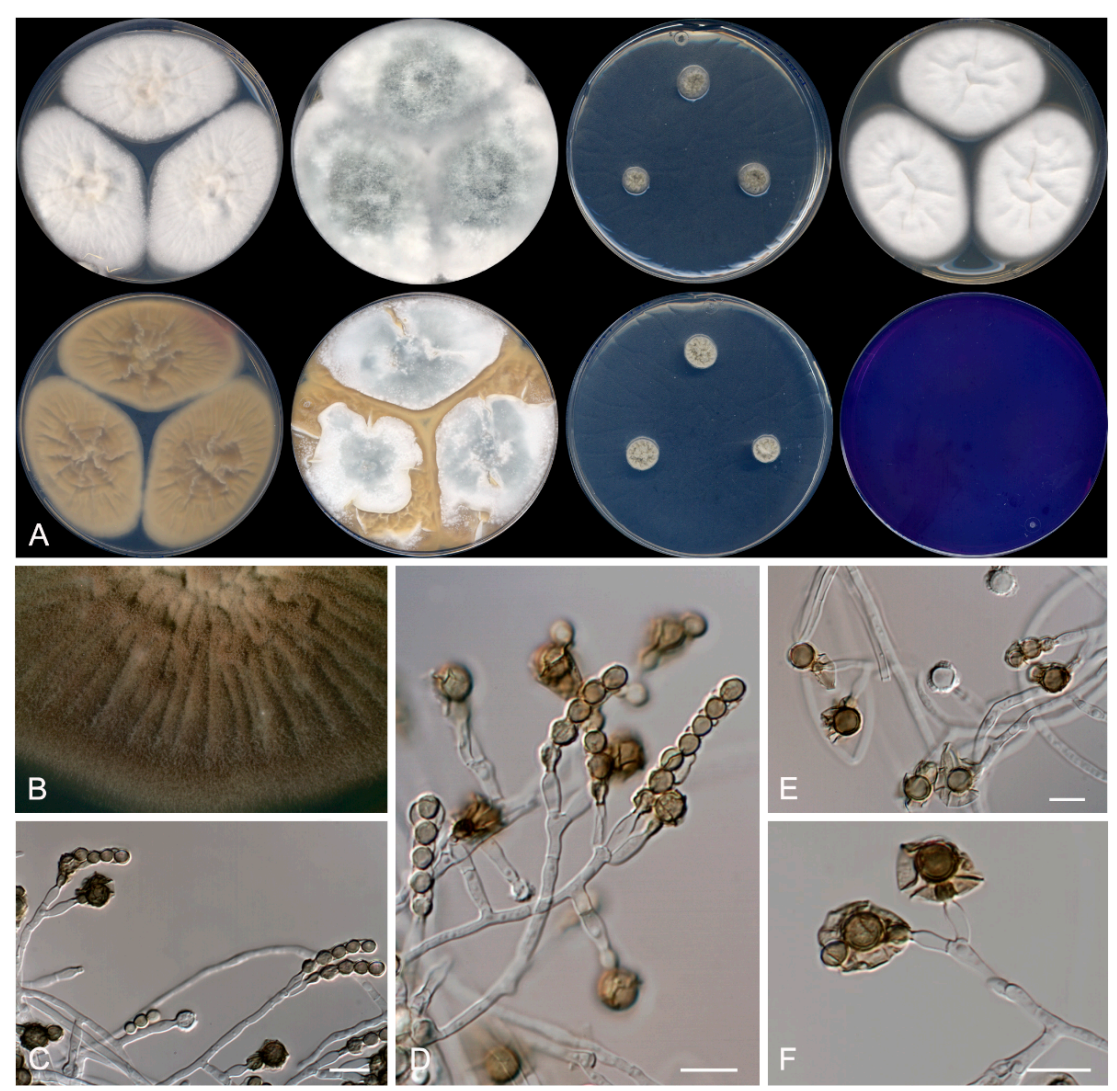
Morphological features of *Penicillium melanosporum* FMR 17,424 ^T^. (**A**) Colonies after 14 days at 25 °C (top row, left to right: surface on Czapek yeast extract agar (CYA), yeast extract sucrose agar (YES), dichloran^®^ 18% glycerol agar (DG18), and malt extract agar (MEA); bottom row, left to right: reverse on CYA, YES and DG18, and surface on creatine sucrose agar (CREA)). (**B**) Detail of the colony on CYA under the stereomicroscope. (**C**–**F**) Conidiophores. Scale Bar = 10 μm.

*Penicillium melanosporum* Rodr.-Andr., Cano and Stchigel, *sp. nov*. MycoBank MB 835938 (Figure 2).

Etymology: From Greek *μελανό*-, black, and -*σπόριο*, spore, referring to the production of dark-pigmented conidia.

Section Lanata-Divaricata.

Type: Spain, Castilla y León, Riaza, from a soil sample, 12 May 2018, E. Rodríguez-Andrade and J. F. Cano (holotype CBS H-24465, cultures ex-type FMR 17,424 = CBS 146938).

Description: Mycelium composed of hyaline, septate, smooth- and thin-walled hyphae, 2–4 μm wide. Conidiophores divaricate, monoverticillate or reduced to a single phialide arising directly from the vegetative hyphae; stipes hyaline, non- to 1–2-septate, mostly not septate at the base, smooth- and thin-walled, 15–70 × 2–3 μm; phialides 1 to 3 at the top of the stipe, hyaline, smooth- and thin-walled, ampuliform with a ventricose base, 9–10 × 2.5–3 μm, sometimes with a dark-colored collarette when old; conidia at first subhyaline becoming olive-green to dark brown with the age, smooth-walled to verruculose, subglobose, 4–5 μm, several of them covered by a dark brown sheath with the age, the last of the conidia produced remaining attached to the phialide, thick-walled and coarsely ornamented, globose, 6–7 μm of diameters, always surrounded by a dark brown sheath. Sclerotia not produced. Sexual morph not observed.

Culture characteristics (14 days at 25 °C): Colonies on CYA reaching 60–65 mm diameters, raised at the center, velvety, sulcate, margins regular, yellowish-white (4A2), sporulation absent to sparse, exudates absent; reverse olive-brown (4D4), soluble pigment absent. On MEA reaching 53–55 mm diameters, slightly raised, velvety to floccose, whitish, sulcate, margins regular, sporulation sparse, exudates absent; reverse light yellow (4A4), soluble pigment absent. On YES reaching 68–71 mm diameters, raised and fluffy, margins regular, grey (24D1) at the center and white (4A1) at the edge, sporulation abundant, exudates absent; reverse brownish orange (5C4), soluble pigment absent. On OA reaching 65–68 mm diameters, slightly raised, floccose and fluffy, whitish with turquoise grey (24D2) spots, sporulation abundant, exudates absent; soluble pigment absent. On DG18 reaching 8–11 mm diameters, raised, olive-brown (4D4) and yellowish-grey (4B2), margins regular, sporulation sparse, exudates absent; reverse greyish yellow (3C3) at the center and yellowish-white (4A2) at the edge, soluble pigment absent. On G25N reaching 25–26 mm diameters, slightly elevated, cottony, sulcate, margins regular, white (4A1) at the center, and greyish beige (4C2) at the edge, sporulation abundant, exudates absent; reverse greyish yellow (4C5), soluble pigment absent. On MY70FG reaching 22–24 mm diameters, slightly elevated, sulcate, cerebriform, margins regular, greyish orange (5B3), sporulation sparse, exudates absent; reverse orange white (5A2), soluble pigment absent. On CREA reaching 58–59 mm diameters, flattened, slightly powdery, whitish, exudates absent, sporulation sparse, margins irregular; no acid production. Cardinal temperatures of growth: minimum 15 °C, optimum 30 °C, maximum 40 °C.

Barcodes: ITS barcode LR655192 (alternative markers: *BenA* = LR655196; *CaM* = LR655200; *rpb*2 = LR655204).

Notes: *Penicillium meloforme* and *Penicillium siccitolerans* sp. nov., which form a well-supported terminal clade in our tree (Figure 1), are the species most phylogenetically related to *P. melanosporum*. *Penicillium melanosporum* differs from *P. meloforme*, because the former produces an asexual morph and lacks a sexual morph, while the second one forms a sexual morph and the asexual morph is only produced on MY70FG. *Penicillium siccitolerans* differs from *P. melanosporum* by the production of sclerotia. *Penicillium melanosporum* also produces shorter stipes than those of *P. meloforme* (15–70 × 2–3 μm versus 150–500 × 2–3 μm) [29], and bigger conidia (4–5 μm diameters) than those of *P. meloforme* (2–3 × 1–2.5 μm) and of *P. siccitolerans* (2–3 × 1–2.5 μm). Moreover, *P. melanosporum* differs from *P. meloforme* and *P. siccitolerans* by the production of a mucilaginous brown to dark brown exopigment surrounding the conidia, and because the last of the conidia produced remains attached to the phialide. Nevertheless, *P. melanosporum* and *P. siccitolerans* are capable to grow on CYA at 40 °C, while *P. meloforme* does not grow at 37 °C [29].

**Figure 3:**
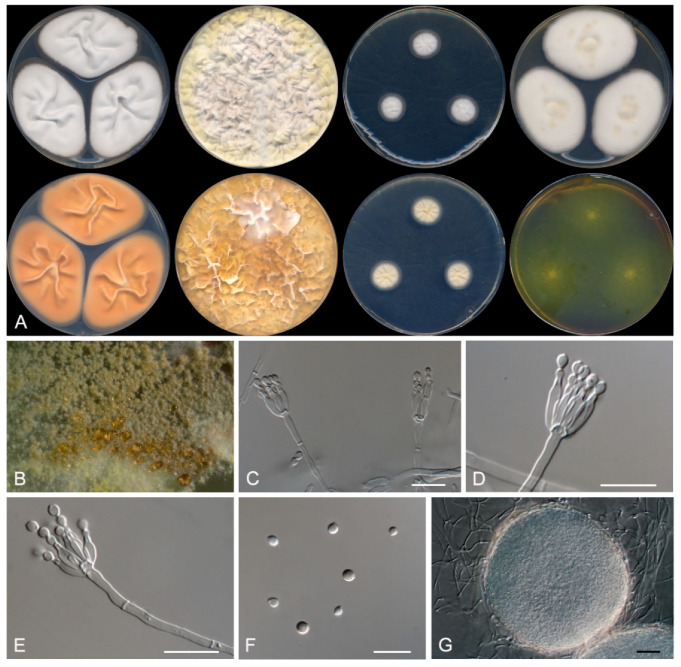
Morphological characters of *Penicillium siccitolerans* FMR 17,381 ^T^. (**A**) Colonies after 14 days at 25 °C (top row, left to right: surface on CYA, YES, DG18, and MEA; bottom row, left to right: reverse on CYA, YES and DG18, and surface on CREA). (**B**) Detail of the colony on oatmeal agar (OA) under the stereomicroscope. (**C**–**E**) Conidiophores. (**F**) Conidia. (**G**) Sclerotium. Scale Bar: (**C**–**F**) = 10 μm; G = 25 μm.

*Penicillium siccitolerans* Rodr.-Andr., Stchigel and Cano, *sp. nov.* MycoBank MB 835939. Figure 3.

Etymology: From Latin *siccus*-, dry, and -*tolerans*, tolerance, due to the ability of this fungus to grow at a low water activity.

Section Lanata-Divaricata.

Type: Spain, Castilla y León, Riaza, from a soil sample, 12 May 2018, E. Rodríguez-Andrade and J. F. Cano (holotype CBS H-24466, cultures ex-type FMR 17381).

Description: Mycelium superficial to immersed composed of hyaline, septate, smooth-walled hyphae, 1.5–2 μm wide. Conidiophores monoverticillate; stipes smooth-walled, 30–80 × 1.5–3 μm; phialides (1) 2–6 (–10) per stipe, ampuliform to flask-shaped, smooth-walled, 9–11 × 1.5–2 μm; conidia smooth-walled, olive green when mature, broadly limoniform, 2.5–3.5 × 2.5–3 μm. Sclerotia white, translucent, slightly tomentose, mostly globose, 120–190 μm diameters, composed of hyaline, thin-walled, polygonal cells of 5–8 μm diameters Sexual morph not observed.

Culture characteristics (14 days at 25 °C): Colonies on CYA reaching 59–61 mm diameters, flattened, sulcate, with regular margins, white (5A1), sporulation sparse, hyaline exudates scarce; reverse greyish orange (5B5), soluble pigment absent. On MEA reaching 47–50 mm diameters, slightly raised, velvety, margins regular, yellowish-white (4A2), sporulation absent, exudates absent; reverse light yellow (4A5), soluble pigment absent. On YES reaching 65–69 mm diameters, raised sulcate, margins regular, white (4A1) at the center and pastel yellow (2A4) at the edge, sporulation sparse, exudates absent; reverse orange (5A6), soluble pigment absent. On OA reaching 73–75 mm diameters, flattened, granulose, margins irregular, olive-brown (4D4) at the center and pale yellow (3A3) at the edge, sporulation sparse, exudates dark yellow (4C8); soluble pigment absent. On DG18 reaching 13–16 mm diameters, flattened, white (4A1), margins regular, sporulation sparse, exudates absent; reverse yellowish-white (3A2), soluble pigment absent. On G25N reaching 25–26 mm diameters, slightly elevated, velvety, margins regular, white (4A1) with reddish-yellow (4A6) spots, sporulation sparse, exudates absent; reverse orange (5A7), soluble pigment absent. On MY70FG reaching 24–25 mm diameters, slightly elevated, velvety, regular margins, white (1A1), sporulation sparse, exudates absent; reverse pale-yellow (4A3), soluble pigment absent. On CREA reaching 49–53 mm diameters, flattened, granulose, margins irregular, white (4A1), sporulation sparse, exudates absent; acid production weak. Cardinal temperatures of growth: minimum 15 °C, optimum 30 °C, maximum 40 °C.

Barcodes: ITS barcode LR655193 (alternative markers: *BenA* = LR655197; *CaM* = LR655201; *rpb*2 = LR655205).

Notes: *Penicillium siccitolerans* is phylogenetically close related to *P. meloforme*. Nevertheless, *P. siccitolerans* does not produce a sexual morph, which is present in *P. meloforme*. For additional differences between these species, see *P. melanosporum* notes (above).

**Figure 4:**
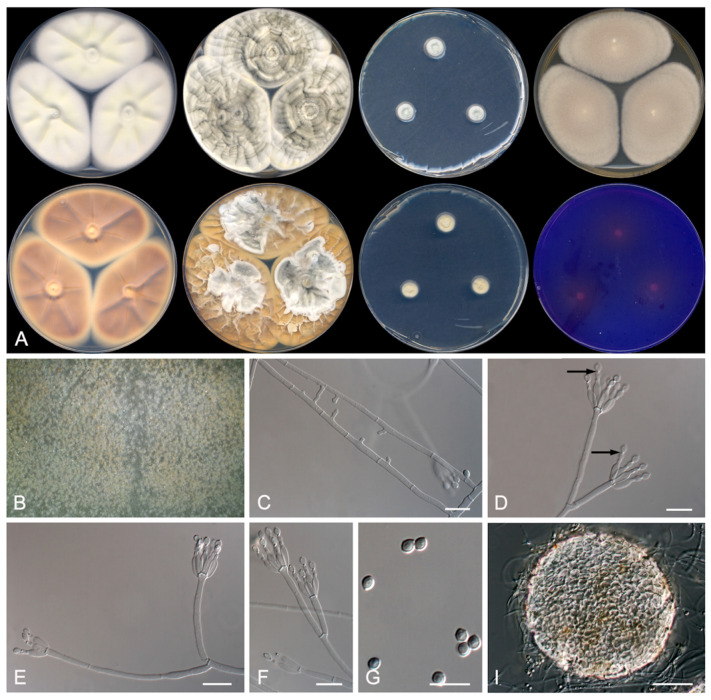
Morphological characters *Penicillium michoacanense* FMR 17,612 ^T^. (**A**) Colonies after 14 days at 25 °C (top row, left to right: surface on CYA, YES, DG18, and MEA; bottom row, left to right: reverse on CYA, YES and DG18, and surface on CREA). (**B**) Detail of the colony on OA under the stereomicroscope. (**C**) Anastomosing hyphae. (**D**–**F**). Conidiophores (arrows showing disjunctors between conidia). (**G**) Conidia. I. Sclerotium. Scale Bar: (**C**–**G**) = 10 μm; I = 50 μm.

*Penicillium michoacanense* Rodr.-Andr., Cano and Stchigel, *sp. nov.* MycoBank MB 835940. Figure 4.

Etymology: The species name refers to Michoacán state, México, the geographical area where the soil sample was collected.

Section Lanata-Divaricata.

Type: México, Michoacán state, Jiménez, El Zapote, from soil intended for corn cultivation, 03 January 2010, E. Rodríguez and A. González (holotype CBS H-24467, cultures ex-type FMR 17612).

Description: Mycelium superficial to immersed composed of septate, anastomosing, smooth-walled, hyaline hyphae of 1–2 μm wide. Conidiophores divaricate, monoverticillate; stipes hyaline, smooth–walled, 15–60 × 1–1.5 μm; phialides (1–) 4–6 (7) per stipe, hyaline, smooth–walled, flask-shaped, 4–5 × 1.5 μm; conidia olive green, mostly smooth-walled with a few warts, broadly limoniform, 1.5–2.5 × 1–2 μm, separated by conspicuous disjunctors. Sclerotia hyaline to light brown, globose, 70–120 × 90–140 μm, composed by very thick–walled, highly refractive, polyhedral to globose cells of 5–12 μm diameters Sexual morph not observed.

Culture characteristics (14 days at 25 °C): Colonies on CYA reaching 62–64 mm diameters, flattened, margins regular, pastel yellow (1A4) at the center and white (1A1) at the edge, sporulation sparse, with a little production of hyaline exudates; reverse yellowish brown (5D5), soluble pigment absent. On MEA reaching 55–57 mm diameters, flattened, velvety, margins regular, yellowish grey (3C2), sporulation sparse, exudates absent; reverse greyish yellow (4B5), soluble pigment absent. On YES reaching 63–67 mm diameters, raised, sulcate, velvety to floccose, margins regular, brownish grey (4D2) at the center and white (4D1) with pastel yellow (3A4) spots at the edge, sporulation abundant, with a little production of brownish orange (5C6) exudates; reverse, greyish orange (5B4), soluble pigment absent. On OA reaching 69–70 mm diameters, flattened, granulose, margins irregular, brownish grey (5D2) at the center and greyish yellow (4B3), sporulation abundant, exudates production pale yellow (4A3); soluble pigment absent. On DG18 reaching 10–11 mm diameters, slightly raised, margins regular, velvety to floccose, greenish grey (26B2) at the center and white (6A1) at the edge, sporulation abundant, exudates absent; reverse yellowish white (4A2), soluble pigment absent. On G25N reaching 23–24 mm diameters, slightly elevated, velvety, margins regular, yellowish white (4A2) at the center, then orange-grey (5B2) and turquoise-white (24A2) at the edge, sporulation sparse, exudates absent; reverse pale-yellow (4A3), soluble pigment absent. On MY70FG reaching 25–27 mm diameters, slightly elevated, sulcate, velvety, margins regular, white (1A1), exudates absent, sporulation sparse; reverse light-yellow (4A4), soluble pigment absent. On CREA reaching 25–26 mm diameters, flattened, granulose, margins irregular, yellowish white (3A2), sporulation abundant, exudates absent; soluble pigment absent, acid production weak. Cardinal temperatures of growth: minimum 15 °C, optimum 30 °C, maximum 37 °C.

Barcodes: ITS barcode LR655194 (alternative markers: *BenA* = LR655198; *CaM* = LR655202; *rpb*2 = LR655206).

Notes: *Penicillium michoacanense* is phylogenetically close to *P. limosum*. Nevertheless, *P. michoacanense* has shorter and thinner stipes (15–60 × 1–1.5 μm) and smaller conidia (1.5–2.5 × 1–2 μm) than those of *P. limosum* ((62–) 75–225 × 2–3 (–3.5) μm, and 2.8–3.3 × 2.5–3 μm, respectively) [30]. In addition, *P. michoacanense* does not produce the sexual morph on any media tested, which is produced by *P. limosum* on MEA and OA [30].

**Figure 5:**
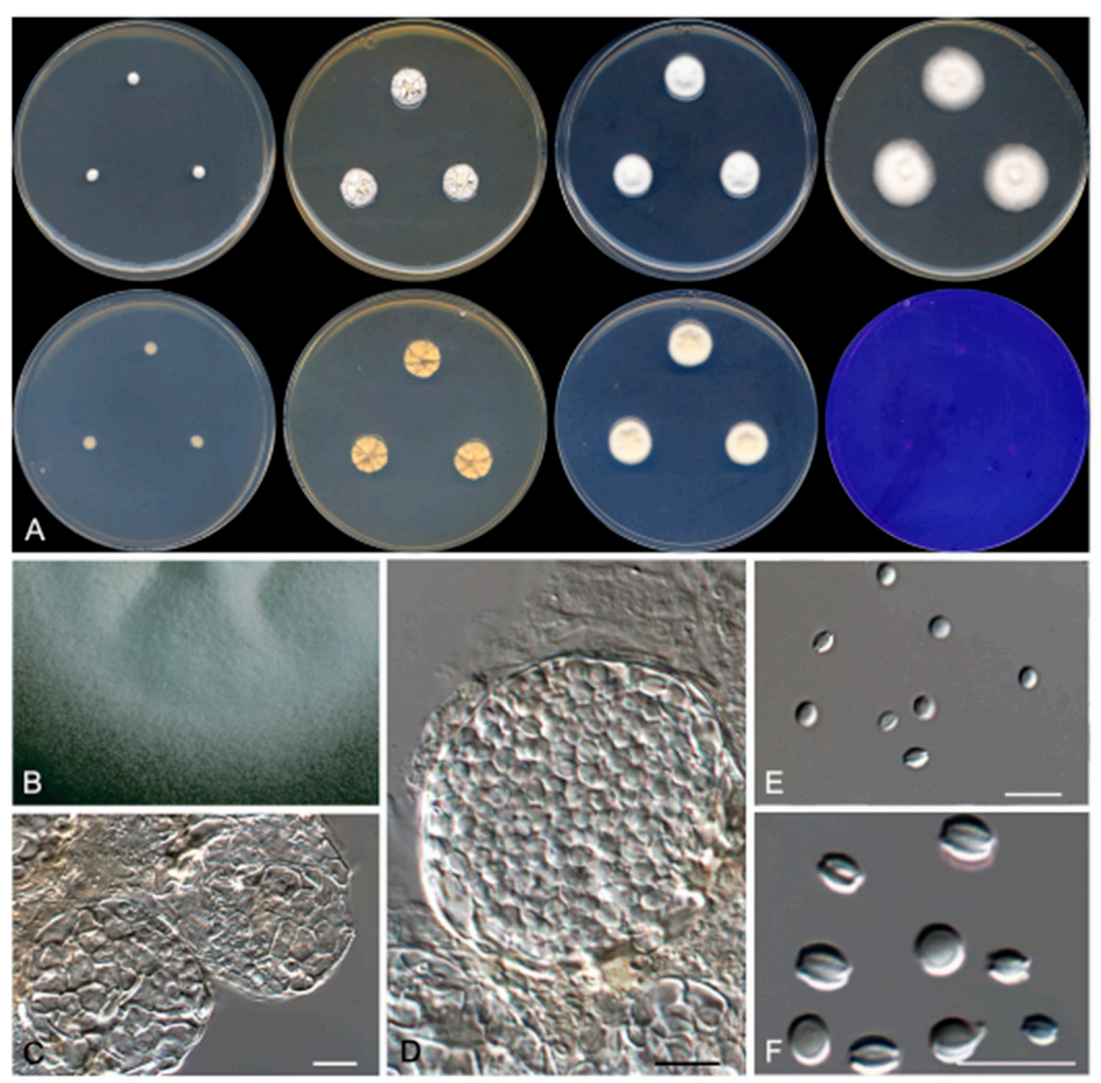
Morphological characters *Penicillium sexuale* FMR 17,380 ^T^. (**A**) Colonies after 14 days at 25 °C (top row, left to right: surface on CYA, YES, DG18, and MEA; bottom row, left to right: reverse on CYA, YES, DG18, and surface on CREA). (**B**) Colony on MEA under the stereomicroscope. (**C**,**D**) Ascomata. (**E**,**F**) Ascospores. Scale Bar = 10 μm.

*Penicillium sexuale* Rodr.-Andr., Stchigel and Cano, *sp. nov.* MycoBank MB 835941. Figure 5.

Etymology: Referring to the fact that only presents a sexual morph.

Section *Crypta*.

Type: Spain, Castilla y León, Riaza, from a soil sample, 12 May. 2018, E. Rodríguez-Andrade and J. F. Cano (holotype CBS H-24468, cultures ex-type FMR 17380 = CBS 146939).

Description: Mycelium superficial to immersed composed of septate, smooth-walled, hyaline hyphae of 1–2 μm wide. Ascostromata cream to tan-colored, more or less globose, 30–50 μm diameters, composed of translucent, refringent, thick-walled polygonal cells of 5–12 μm diam, peridial wall becoming 1–2-layered when the ascospores are produced, outer peridial layer of textura angulata. Asci 8-spored, borne singly, globose, 7–10 μm diameters Ascospores hyaline, smooth-walled under the brightfield microscope, broadly lenticular, 2.5–3 × 2–2.5 μm, with two widely separated equatorial ridges and with an equatorial furrow. Asexual morph not observed.

Culture characteristics (14 days at 25 °C): Colonies on CYA reaching 4–5 mm diameters, slightly raised, velvety, margins regular, white (4A1), exudates absent, sporulation absent; reverse yellowish-white (4A2), soluble pigment absent. On MEA reaching 21–22 mm diameters, slightly raised, velvety, margins regular, white (3A1), sporulation absent, exudates absent; reverse pale yellow (4A3), soluble pigment absent. On YES reaching 11–12 mm diameters, raised, sulcate, velvety, margins regular, yellowish-white (4A2), sporulation absent, exudates absent; reverse pastel yellow (4A4), soluble pigment absent. On DG18 reaching 14–15 mm diameters, slightly raised, velvety, margins regular, white (4A1), sporulation absent, little production of hyaline exudates; reverse pale yellow (4A3), soluble pigment absent. On G25N reaching 5–6 mm diameters, slightly elevated, velvety, margins regular, white (4A1), sporulation absent, exudates absent; reverse pale yellow (4A3), soluble pigment absent. On MY70FG reaching 6–8 mm diameters, slightly elevated, velvety, margins regular, white (1A1), exudates absent, sporulation absent; reverse pale-yellow (4A3), soluble pigment absent. On CREA reaching 4–5 mm diameters, flattened, velvety to floccose, margins irregular, white (4A1), sporulation absent, exudates absent; acid production absent. Cardinal temperatures of growth: minimum 15 °C, optimum 25 °C, maximum 37 °C.

Barcodes: ITS barcode LR655195 (alternative markers: *BenA* = LR655199; *CaM* = LR655203; *rpb*2 = LR655207).

Notes: *Penicillium sexuale* differs significantly from *P. cryptum* [31], the phylogenetically nearest species (see Figure 1), by a very a late production of ascospores into the ascostromata (after 2 months growing on PDA; after two weeks in *P. cryptum*), and because does not produce an asexual morph in any of the culture media tested.

## 4. Discussion

Xerophilic fungi are those able to grow at a water activity (a_w_) of 0.85 or below [12]. The order Eurotiales, which contains the genus *Penicillium*, includes several xerophilic genera. Among them, one of the best characterized is *Aspergillus.* This genus has many species of xerophilic habit, mostly foodborne but also inhabitants of soil, able to grow at a_w_ of up to 0.67 [32,33]. Other eurotialean xerophilic genera are *Monascus* [34,35,36], *Talaromyces* [10], and *Xerochrysium* [37]. However, the most xerophilic of the fungal taxa is *Xeromyces bisporus*, able to grow at a_w_ between 0.61–0.62 [38].

There are a large number of published studies on xerophilic species and on the physiological mechanisms involved for the genus *Aspergillus*, but conversely very few studies on species of *Penicillium*. Our findings strongly suggest that more studies are needed for a better understanding of the diversity of extremophilic species of *Penicillium*, and the mechanisms involved in their adaptation to extreme environments.

Species of *Penicillium* living in soils, caves, and buildings, and causing food spoilage, such as *P. brevicompactum*, *P. chrysogenum*, *P. cinnamopurpureum*, *P. implicatum*, and *P. janczewskii* grow at a minimum a_w_ of 0.78; *Penicillium corylophilum*, *P. fellutanum*, *P. viridicatum*, and *P. verrucosum* develop at a_w_ as low 0.80; and *Penicillium aurantiogriseum*, *P. citrinum*, *P. expansum*, *P. griseofulvum*, and *P. restricum* grow at a_w_ of 0.81–0.82 [12,39,40,41,42,43,44]. As these species grow at a_w_ lower than 0.85, all of them must be considered as xerophilic [43]. Very recently, *Penicillium apimei*, *P. meliponae*, and *P. mellis* have been described in honey produced by stingless bees in Brazil [10]. Despite these species were isolated from a sugar-rich substrate, with a_w_ usually lower than 0.60, and being included in other recent taxonomic studies on *Penicillium* species, the ability to grow at low water activity was not tested.

In the present study, the multigene-based phylogeny (using ITS, *BenA*, *CaM*, and *rpb*2 sequences) allowed us initially to describe four new species of *Penicillium* isolated from soil samples collected from Spain and Mexico—*P*. *melanosporum*, *P. michoacanense*, *P. siccitolerans* of the section *Lanata-Divaricata*, and *P. sexuale*, of the section *Crypta*. All four *Penicillium* spp. were able to grow at a_w_ of 0.76 (on MY70FG culture medium) formed colonies, thus demonstrating they were xerophilic. The asexual morph of *P. melanosporum* resembles those of *P. brunneoconidiatum* and *P. tsitsikammaense* [45] of the section *Aspergilloides*. However, the strongly ornamented dark brown conidia were only observed in the latter, produced by the phialides in *P. melanosporum*, all conidia being equally ornamented in the other two species. The asexual morph of *P. michoacanense* and *P. siccitolerans* is also reminiscent of the species of the genus within the section *Aspergilloides* [45] than those of the section *Lanata-Divaricata*. *P. michoacanense* and *P. siccitolerans* produce sclerotia, but these are made up of polygonal cells that are thick-walled in the former and thin-walled in latter. Finally, *P. sexuale* differs from *P. cryptum* because it does not form the typical asexual morph consisting in (mostly) conidiophores with solitary phialides, presenting only a sexual morph.

## 5. Conclusions

In the present study, we report the isolation of four new xerophilic species of the genus *Penicillium*, all of them isolated from soil samples. Three of these species were placed in the section *Lanata-Divaricata* (*P. melanosporum*, *P. michoacanense*, and *P. siccitolerans*), and the fourth in section *Crypta* (*P. sexuale*). Our study shows that there are metabolic groups still to be explored within the genus *Penicillium*, and that known species need to be characterized physiologically in depth, such as species of the genus *Aspergillus*.

## Data Availability

Not applicable.

## Supplementary Materials

The following are available online at https://www.mdpi.com/2309-608X/7/2/126/s1, Table S1: *Penicillium* spp. sequences used in this study.
