# Supplementary material for: New Xerophilic Species of Penicillium from Soil
Source: J Fungi (Basel). 2021 Feb 9;7(2):126. doi: 10.3390/jof7020126 (PMC7915375; doi:10.3390/jof7020126)
Supplement: Supplementary file 1 [file jof-07-00126-s001.pdf]

Supplementary Table S1. *Penicillium* spp. sequences used in this study.

Species name	Strains	GenBank accession number			
		ITS	*BenA*	*CaM*	*rpb2*
Penicillium abidjanum	CBS 246.67 = ATCC 18385 = FRR 1156 = IMI 136244 ^{T}	GU981582	GU981650	KF296383	JN121469
Penicillium aeris	CBS 135897 = DTO 207D4 ^{T}	KF303654	KF303614	KF303627	KF303681
Penicillium alagoense	URM 8086 ^{T}	MK804503	MK802333	MK802336	MK802338
Penicillium alfredii	CBS 138224 = DTO 269-A4 ^{T}	KJ775684	KJ775177	KJ775411	KJ834520
Penicillium amphipolaria	DAOMC 250551 = CBS 140997 ^{T}	KT887872	KT887833	KT887794	-
Penicillium annulatum	CBS 135126 = DTO 180-G7 ^{T}	JX091426	JX091514	JX141545	KF296410
Penicillium araracuaraense	CBS 113149 = IBT 23247 ^{T}	GU981597	GU981642	KF296373	KF296414
Penicillium austricola	CBS 135900 = DTO 183E6 = DAOM 241066 ^{T}	JX091466	JX091579	JX141600	KF303705
Penicillium austrosinense	CGMCC 3.18797 = CBS 144505 ^{T}	KY495007	KY495116	KY494947	KY495061
Penicillium bissettii	DAOMC 167011 = CBS 140972 ^{T}	KT887845	KT887806	KT887767	-
Penicillium brasilianum	CBS 253.55 = ATCC 12072 = FRR 3466 ^{T}	GU981577	GU981629	AB667857	KF296420
Penicillium brefeldianum	CBS 235.81 = NRRL 710 = FRR 710 = IFO 31731 = IMI 216896 ^{T}	AF033435	GU981623	AB667857	KF296421
Penicillium camponotum	DAOMC 250557 = CBS 140982 ^{T}	KT887855	KT887816	KT887777	-
Penicillium cantabricum	CBS 120415 = DTO 76I9 = FMR 9121 ^{T}	KF303655	KF303615	KF303646	KF303682
Penicillium caperatum	CBS 443.75 = ATCC 28046 ^{T}	KC411761	GU981660	KF296392	KF296422

Penicillium catalonicum	CBS 110532 = DTO 78H5 [T]	KF303650	KF303609	KF303644	KF303683
Penicillium cataractum	DAOMC 250534 = CBS 140974 [T]	KT887847	KT887808	KT887769	-
Penicillium cluniae	CBS 326.89 [T]	KF296406	KF296471	KF296402	KF296424
Penicillium coeruleum	CBS 141.45 [T]	GU981606	GU981655	KF296393	KF296425
Penicillium corylophilum	CBS 330.79 [T]	GU944557	GU944519	GU944607	JN406569
Penicillium cremeogriseum	CBS 223.66 = ATCC 18320 = ATCC 18323 = FRR 1734 = IMI 197492 = NRRL 3389 [T]	GU981586	GU981624	KF296403	KF296426
Penicillium cryptum	CBS 271.89 = DTO 122C9 = ATCC 60138 = IMI 296794 = NRRL 13460 [T]	KF303647	KF303608	KF303628	JN121478
Penicillium curticaule	CBS 135127 = DTO 180-D3 = DAOM 241159 [T]	FJ231021	JX091526	JX141536	KF296417
Penicillium daleae	CBS 211.28 = ATCC 10435 = FRR 2025 = IFO 6087 = IFO 9072 [T]	GU981583	GU981649	KF296385	KF296427
Penicillium diatomitis	CBS 140107 = IBT 30728 [T]	FJ430748	HE651133	LT970912	LT797560
Penicillium ehrlichii	CBS 324.48 = ATCC 10442 = IMI 039737 = NRRL 708 [T]	AF033432	GU981652	KF296395	KF296428
Penicillium elleniae	CBS 118135 = IBT 23229 [T]	GU981612	GU981663	KF296389	KF296429
Penicillium excelsum	ITAL7572 = IBT 31516 [T]	KR815341	KP691061	KR815342	-
Penicillium flaviroseum	CGMCC 3.18805 = CBS 144479 [T]	KY495032	KY495141	KY494972	KY495083
Penicillium fructuariae-cellae	CBS 145110 [T]	MK039434	KU554679	MK045337	-
Penicillium glaucoroseum	CBS 138908 = NRRL 908 (Not type) [T]	KF296407	KF296469	KF296400	KF296430
Penicillium globosum	CGMCC 3.18800 = CBS 144639 [T]	KY495014	KY495123	KY494954	KY495067
Penicillium griseoflavum	CGMCC 3.18799 = CBS 144525 [T]	KY495011	KY495120	KY494951	KY495064

Penicillium griseopurpureum	CBS 406.65 = ATCC 22353 = FRR 3429 = IFO 9147 = IMI 096157 [T]	KF296408	KF296467	KF296384	KF296431
Penicillium guaibinense	CCDCA 11512 [T]	MH674389	MH674391	MH674393	-
Penicillium guangxiense	CGMCC 3.18793 = CBS 144526 [T]	KY494986	KY495095	KY494926	-
Penicillium hainanense	CGMCC 3.18798 = CBS 144527 [T]	KY495009	KY495118	KY494949	-
Penicillium infrabuccalum	DAOMC 250537 = CBS 140983 [T]	KT887856	KT887817	KT887778	-
Penicillium janthinellum	CBS 340.48 = ATCC 10455 = IMI 040238 = NRRL 2016 [T]	GU981585	GU981625	KF296401	JN121497
Penicillium javanicum	CBS 341.48 = ATCC 9099 = IFO 31735 = IMI 039733 = MUCL 29099 = NRRL 707 [T]	GU981613	GU981657	KF296387	JN121498
Penicillium jianfenglingense	CGMCC 3.18802 = CBS 144640 [T]	KY495016	KY495125	KY494956	KY495069
Penicillium koreense	KACC 47721 [T]	KJ801939	KM000846	-	-
Penicillium laevigatum	CGMCC 3.18801 = CBS 144481 [T]	KY495015	KY495124	KY494955	KY495068
Penicillium lagena	CBS 185.65 = DTO 77I8 = MUCL 8221 [T]	KF303665	KF303619	KF303634	JN121450
Penicillium lassenii	CBS 277.70 = DTO 95D6 = NRRL 5272 = ATCC 22054 = FRR 858 = IMI 148395 [T]	KF303648	KF303607	KF303629	JN121481
Penicillium levitum	CBS 345.48 = ATCC 10464 = IFO 6101 = IFO 8849 = IMI 039735 [T]	GU981607	GU981654	KF296394	KF296432
Penicillium limosum	CBS 339.97 [T]	GU981568	GU981621	KF296398	KF296433
Penicillium lineolatum	CBS 188.77 [T]	GU981579	GU981620	KF296397	KF296434
Penicillium ludwigii	CBS 417.68 = FRR 559 [T]	KF296409	KF296468	KF296404	KF296435
Penicillium malacosphaerulum	CBS 135120 = DTO 180-E6 = DAOM 241161 [T]	FJ231026	JX091524	JX141542	KF296438

Penicillium mariae-crucis	CBS 271.83 = IMI 256075 [T]	GU981593	GU981630	KF296374	KF296439
Penicillium marthae-christenseniae	CBS 129213 = DTO 201B5 [T]	KF303651	KF303613	KF303645	KF303711
Penicillium melanosporum	**FMR 17424 [T]**	**LR655192**	**LR655196**	**LR655200**	**LR655204**
Penicillium meloforme	CBS 445.74 = ATCC 28049 = IMI 216903 [T]	KC411762	GU981656	KF296396	KF296440
Penicillium michoacanense	**FMR 17612 [T]**	**LR655194**	**LR655198**	**LR655202**	**LR655206**
Penicillium ochrochloron	CBS 357.48 = ATCC 10540 = IMI 039806 = NRRL 926 [T]	GU981604	GU981672	KF296378	KF296445
Penicillium onobense	CBS 174.81 = ATCC 42225 [T]	GU981575	GU981627	KF296371	KF296447
Penicillium oregonense	CBS 129775 = DTO 208A5 [T]	KF303668	KF303623	KF303640	KF303710
Penicillium ortum	CBS 135669 = DTO 180-I9 [T]	JX091427	JX091520	JX141551	KF296443
Penicillium oxalicum	CBS 219.30 = ATCC 1126 = FRR 787 = IMI 192332 = MUCL 29047 [T]	AF033438	KF296462	KF296367	JN121456
Penicillium panissanguineum	DAOMC 250562 = CBS 140989 [T]	KT887862	KT887823	KT887784	-
Penicillium paraherquei	CBS 338.59 = ATCC 22354 = ATCC 46903 = FRR 3454 = IFO 6234 = IMI 068220 = NRRL 3454 [T]	AF178511	KF296465	KF296372	KF296449
Penicillium pedernalense	CBS 140770 [T]	KU255398	KU255396	-	-
Penicillium penarojense	CBS 113178 = IBT 23262 [T]	GU981570	GU981646	KF296381	KF296450
Penicillium piscarium	CBS 362.48 = ATCC 10482 = FRR 1075 = IFO 8111 = IMI 040032 [T]	GU981600	GU981668	KF296379	KF296451
Penicillium porphyreum	CBS 382.64 = DTO 78G7 [T]	KF303666	KF303621	KF303636	KF303677

Penicillium pulvillorum	CBS 280.39 = IFO 7763 = NRRL 2026 [T]	AF178517	GU981670	KF296377	KF296452
Penicillium raperi	CBS 281.58 = ATCC 22355 = IFO 8179 = IMI 071625 = NRRL 2674 [T]	AF033433	GU981622	KF296399	KF296453
Penicillium restrictum	CBS 367.48 = ATCC 11257 = FRR 1748 = IMI 040228 = NRRL 1748 [T]	AF033457	KJ834486	KP016803	JN121506
Penicillium reticulisporum	CBS 122.68 = ATCC 18566 = IFO 9024 = IMI 136700 [T]	AF033437	GU981665	KF296391	KF296454
Penicillium riverlandense	CBS 135896 = DTO 182F6 = DAOMC 241060 [T]	JX091457	JX091580	JX141593	KF303685
Penicillium rolfsii	CBS 368.48 = ATCC 10491 = FRR 1078 = IFO 7735 [T]	JN617705	GU981667	KF296375	KF296455
Penicillium rubriannulatum	CGMCC 3.18804 = CBS 144641 [T]	KY495029	KY495138	KY494969	KY495080
Penicillium setosum	CBS 144865 [T]	KT852579	MF184995	MH105905	-
Penicillium sexuale	**FMR 17380 [T]**	**LR655195**	**LR655199**	**LR655203**	**LR655207**
Penicillium siccitolerans	**FMR 17381 [T]**	**LR655193**	**LR655197**	**LR655201**	**LR655205**
Penicillium simplicissimum	CBS 372.48 = ATCC 10495 = FRR 902 = IFO 5762 = IMI 039816 [T]	GU981588	GU981632	KF296368	JN121507
Penicillium singorense	CBS 138214 = DTO 133-C6 [T]	KJ775674	KJ775167	KJ775403	-
Penicillium skrjabinii	CBS 439.75 = NRRL 13055 = FRR 1945 = IMI 196528 [T]	GU981576	GU981626	KF296370	EU427252
Penicillium annulatum	CGMCC 3.18806 = CBS 144482 [T]	KY495038	KY495147	KY494978	-
Penicillium soosanum	CBS 140106 = IBT 30727 [T]	FJ430745	FM865811	LT970913	LT797561
Penicillium spinuliferum	CGMCC 3.18807 = CBS 144483 [T]	KY495040	KY495149	KY494980	KY495090
Penicillium subrubescens	CBS 132785 = DTO 188-D6 [T]	KC346350	KC346327	KC346330	KC346306

Penicillium svalbardense	CBS 122416 = IBT 23856 T	GU981603	KC346325	KC346338	KF296457
Penicillium tanzanicum	DAOMC 250514 = CBS 140968 T	KT887841	KT887802	KT887763	-
Penicillium terrarumae	CBS 131811 = DTO 174-H2 T	MN431397	KX650295	MN969323	MN969185
Penicillium toxicarium	NRRL 6172 T	EF198650	EF198620	EF198631	EF198499
Penicillium tubakianum	CBS 287.66 = DTO 138D9 = MUCL 8519 = IFO 8315 T	KF303652	KF303611	KF303637	KF303712
Penicillium vanderhammenii	CBS 126216 = IBT 23203 T	GU981574	GU981647	KF296382	KF296458
Penicillium variratense	CBS 337.97 = DTO 137C8 T	KF303649	KF303610	KF303630	KF303675
Penicillium vasconiae	CBS 339.79 = ATCC 42224 T	GU981599	GU981653	KF296386	KF296459
Penicillium viridissimum	CGMCC 3.18796 = CBS 144484 T	KY495004	KY495113	KY494944	KY495059
Penicillium williamettense	CBS 129774 = DTO 208A4 T	KF303667	KF303622	KF303639	KF303709
Penicillium wisconsinense	CBS 128279 = DTO 198H7 T	KF303670	KF303624	KF303641	KF303706
Penicillium wollemiicola	CBS 137177 = DTO 297E3 T	KJ174314	KJ174315	KJ174316	KJ174313
Penicillium wotroi	CBS 118171 = IBT 23253 T	GU981591	GU981637	KF296369	KF296460
Penicillium yunnanense	CGMCC 3.18794 = CBS 144485 T	KY494990	KY495099	KY494930	KY495048
Penicillium zonatum	CBS 992.72 = ATCC 24353 T	GU981581	GU981651	KF296380	KF296461

[1]**ATCC**: American Type Culture Collection, Virginia, USA; **BCCM/MUCL**: Mycothèque de l'Université catholique de Louvain, Louvain-la-Neuve, Belgium; **CBS**: Culture collection of the Westerdijk Biodiversity Institute, Ultrech, the Netherlands; **CCDCA**: Culture Collection of Microorganims from the Department of Food Science, Brazil; **CGMCC**: China General Microbiological Culture Collection Center, Beijing, China; **DAOM**: Canadian Collection of Fungal Cultures, Ottawa, Canada; **DTO**: Applied and Industrial Mycology Department Collection, Ultrecht, the Netherlands; **FRR**: Food Science Australia, Ryde; **FMR**: Facultad de Medicina, Universitat Rovira i Virgili, Reus, Spain; **IBT**: IBT Culture Collection of Fungi, Lyngby, Denmark; **IFO**: Institute for Fermentation, Osaka, Japan, now NBRC; **IMI**: International Mycological Institute, CABI-Bioscience, Egham, Bakeham Lane, U.K.; **ITAL**: Instituto de Tecnologia de Alimentos, Sao Paulo, Brazil; **KACC**: Korean Agricultural Culture Collection, Suwon, Republic of Korea; **NRRL**: ARS Culture Collection, Peoria, United States; **URM**: Universidade Federal de Pernanbuco, Brazil. **T**: ex-type strain. [2]Sequences newly generated in this study are indicated in **bold**. [3]ITS: internal transcribed spacer region 1 & 2 including 5.8S nrDNA; *BenA*: β-tubulin; *CaM*: calmodulin; *rpb2*: partial RNA polymerase II, second largest subunit.
